# A Plea for More Method in Drug Prescribing

**Published:** 1910-06-11

**Authors:** W. M. Storar


### The General Practitioner's Column.

[Contributions to this Column are invited, and, if accepted, will be paid for.]

### A PLEA FOR MORE METHOD IN DRUG PRESCRIBING.

By W. M. STORAR, L.R.C.P., L.R.C.S.

"The grand aim at once of science and philosophy ought to be the discovery of supreme laws."

The use of drugs as a means towards healing the sick has within recent years fallen very much into disrepute. So much is this the case that it is no uncommon thing to hear even a medical man exclaim, "Ah, I don't believe in physic. There are only four or five drugs I have any confidence in." Unfortunately, this is not merely an individual opinion; it is common in the profession. We so seldom see an article on Therapeutics in our medical journals. Most of the pages are devoted to physiological and pathological disquisitions of very little value to ordinary practitioners—the remaining space being given to minute accounts of new and wonderful surgical procedures.

We are all prepared to admit that in the not very remote past too much reliance was placed on crude drug treatment, and too little attention was given to all the supplementary sciences now included under the terms hygiene and preventive medicine. But while surgery has advanced by leaps and bounds until there is scarcely an organ in the body beyond the reach of the surgeon's knife, medicine has made no corresponding progress. Some say no further progress is possible—that we must only be careful not to interfere unduly with the *vis medicatrix naturæ*, that we may easily do a lot of harm with drugs while it is very questionable whether we can ever do any good. Such therapeutic pessimism is the prevailing professional tone.

Is there really any good excuse for this feeling of hopeless efficiency?

The giving of physic may be only one of the minor and occasional duties of a physician; still most of the drugs in the Pharmacopœia are valuable if used with proper science and discrimination. They are our tools—what we need is greater skill in using them. Hitherto we have had no laws—no rules which have been of any use in helping us to prescribe with any assurance that we should achieve desired results. Every man has been a law unto himself. A new drug very much vaunted one year is derided and discarded the next. There has been no stability anywhere. Now all this is unsatisfactory and disheartening to any man with some love of science, and a desire to do good work.

Consequently, in order to see if there may not be some way out of this seemingly hopeless chaos, let us examine the testimony of a few of the most eminent men in our profession. Where we find them often in agreement, without suspicion of collusion, we may be able, perhaps, to find a reason for such frequent consensus of opinion. A series of seeming coincidences may cause us, upon reflection, to suspect natural law.

As it is necessary to be brief, I shall cursorily examine only seven remedies: Antimony, belladonna, cinchona and quinine, ergot, cantharides, turpentine, and arsenic.

I. Antimony, in the form of Tartar Emetic.—Majendie found in his experiments with animals that it almost always induced inflammatory congestion of the lungs, from which the animals died. Christison, Taylor, and other eminent toxicologists obtained the same results. According to Professor Hughes Bennett, of Edinburgh, when antimony was the routine remedy for pneumonia the mortality was 1 in 5, whereas with no particular treatment, except careful diet and nursing, the mortality was only 1 in 13½. Dr. Pereira naturally remarked that if antimony had a tendency to inflame the lungs, we should expect that large doses of it would not be beneficial in pneumonia. Professor Sir W. T. Gairdner, in his "Clinical Lectures on Pneumonia," says: "In general I regard the ordinary physiological action of antimony as quite opposite to its therapeutic action, and whenever the physiological action occurs, I make it a rule to suspend the remedy or diminish the dose, believing it to be, on the whole, much safer to forego the possible advantage of the antimonial medication than run the risk of superinducing the least degree of poisonous action." He then records a case of pneumonia where he gave tartar emetic to an old, enfeebled, exhausted patient, and says: "In this case, as in several others of similar character which have occurred to me, I ventured, notwithstanding the extreme weakness and exhaustion of the patient, upon the administration of small doses, and was rewarded by seeing the remedy produce its best effects—namely, a therapeutic without the least trace of physiological action. The dose should rarely," he says, "exceed one-tenth or even one-twelfth grain to begin with, sometimes even less." Sir W. Whitla, in pneumonia, recommends "a simple combination" containing, among other things, vinum antimoniale 10 minims—*i.e.* one-twenty-fourth grain—every four hours. Ringer, in the broncho-pneumonia of children, recommends doses of one-sixtieth grain, or even less.

II. Belladona, in a physiological dose produces great dryness of the mouth and tongue extending down the pharynx and larynx, inducing consequently great difficulty in swallowing, together with hoarseness and tickling dry cough.—Dr. John Hardy has employed it in several inflammatory diseases of the throat, and "its good effects are most apparent when the throat and tonsils are acutely inflamed and much swollen." "It is useful to moisten the tongue and control the delirium of fevers." Dr. Handfield-Jones records in the *Lancet* a case of acute tonsillitis, which he cured with belladonna, and he remarks that most of the standard works omit to make any mention of its value in such cases. We know that belladonna will cause active congestion of the brain, with violent excitement, illusions, and delirium. Trousseau and Pidoux, the eminent French clinical teachers, say: "Analogy, that guide so sure in therapeutics, ought to lead us to use belladonna in the treatment of mania, inasmuch as belladonna taken in large doses produces a temporary mania, for experience has proved that a multitude of diseases are cured by therapeutic agents, which seem to act in the same manner as the disease to which we oppose the remedy." Sir W. Whitla says: "Belladonna, stramonium, and hyoscyamus have been used in the treatment of delirium tremens with varying success."

III. Cinchona and Quinine.—Trousseau and Pidoux, quoting Bretonneau, say: "Each day's observation proves that cinchona given in large doses to healthy persons causes in a great number of subjects a very marked febrile condition. The character of this fever, the time when it shows itself, vary in different individuals. There are often tinnitus aurium, deafness, and a species of intoxication preceding the invasion of the fever, a slight shivering then occurs, a dry heat, accompanied by headache, succeeds these first symptoms, which gradually abate and end by sweat." They go on to say that "further doses exceedingly aggravate this fever, which becomes intermittent in type," and they even suggest that many aggravated cases of ague, so-called, are really cases of chronic poisoning with cinchona. Charcot says: "Quinine perseveringly used is sometimes attended with the best results in relieving the vertigo and tinnitus of Menière's disease." Dr. Stephen Mackenzie says he has seen many cases which corroborate this statement (Quain's Dictionary).

IV. Ergot: We know this drug is considered an active uterine ecbolic.—Dr. Alfred Meadows uses the drug in cases of pregnancy during the early weeks of gestation when there is a history of oft-recurring abortion. He further remarks that the dose requires to be carefully regulated, else it may have an effect opposite to what is intended.

Dr. Alfred Freer, of Stourbridge, recorded 200 cases of profuse hæmorrhage with threatened abortion, in which he gave ergot, and he says: "From what I have seen of the use of the remedy I am prepared to maintain that it is a most reliable help, and it often does good, not by forcing the contracting uterus to expel its contents, but often contrary to expectations, by helping the organ to retain its precious charge to its ultimate preservation."

Dr. Lombe Atthill, ex-master of the Rotunda Hospital, recorded a case of threatened abortion which did well under ergot. "From that day on," he says, "I invariably administered ergot to women threatened with abortion. In the majority the threatened symptoms disappeared and pregnancy proceeded normally, but in not one of them did I regret having administered ergot, and I am satisfied that if the ovum is not blighted the ergot acts as a uterine tonic and renders the organ more fitted to undergo the future changes which take place in it during utero-gestation."

V. Cantharides in physiological doses causes acute inflammatory congestion of the kidneys, hæmaturia, cystitis, and strangury.—Sydney Ringer says: "I am convinced of its usefulness in acute Bright's disease after the active fever has subsided." Sir W. Whitla recommends it in minim doses for atony of the bladder, and "with great caution in chronic cystitis." Fredenberg recorded 56 cases of cystitis treated with cantharadin. Of these five bad cases showed no improvement; in 19 cases the improvement was slight; the remaining 32 cases were completely cured, "often," he says, "surprisingly quickly." These cases are quoted by Sir W. Whitla in his Dictionary, apparently with approval. Dr. Octavius Beven, of Balham, detailed a case of hæmaturia in a man of 68 years of age. Dr. Beven had tried all the hæmostatics in the Pharmacopœia without any but prejudicial effect. He was induced to try cantharides. He says: "The first effect of the cantharides was marvellous in that it stopped in 24 hours hæmorrhage which, so far from yielding, had increased under the usual hæmostatics prescribed for a period of ten months." Dr. Goff, of Dundrum, co. Dublin, published a case of hæmaturia which, following Dr. Beven's advice, he treated with cantharides, with the result that in 24 hours, to his astonishment, after taking only four doses, the hæmaturia entirely disappeared and had not recurred some weeks later.

VI. Of Turpentine, whose toxic action is most potent in the kidneys and bladder, Wood, quoting Trousseau, says: "The efficacy of the treatment of chronic catarrh of the bladder by turpentine is such that we may say without rashness that if the wise and well-indicated administration of turpentine does not always cure the malady, it almost always ameliorates the state of the patient."

Drs. Copland and Pereira recommend turpentine in sub-acute consecutive nephritis.

Wood says: "In hæmorrhage from the urinary passages when purely passive or habitual, turpentine is the best hæmostatic that can be used." For chronic Bright's disease Sir W. Whitla says: "It is fashionable to order copaiba, turpentine, and cantharides in infinitesimal doses, which can do no harm."

VII. Arsenic causes great gastric and intestinal pain of a burning character, red tongue, and nausea, vomiting, diarrhœa of a watery type, prostration, cramps in the legs, emaciation, and pallor. Yet Dr. Black, of Chesterfield, speaks of it as practically a specific in cholera even in the stage of collapse. Trousseau advises it in chronic diarrhœa and the diarrhœa of phthisis, also "in extremely small doses" in certain refractory states of the digestive organs—*e.g.* gastro-enteralgia accompanied by obstinate diarrhœa, and in certain cases of lientery, which nothing else can modify." Dr. Warburton Begbie, of Edinburgh, says: "As to an irritable condition of the mucous membrane of the stomach being a bar to its employment, experience has proved that in many cases arsenic is the most valuable remedy we possess for allaying and ultimately removing the morbid condition." For gastralgia, Sir

W. Whitla recommends one-minim doses of liquor arsenicalis before meals. In gastric ulcer he says it relieves pain and checks vomiting. He prescribes it also in nervous diarrhœa, "more so in lienteric diarrhœa and malarious diarrhœa." We are all well acquainted with arsenic as a remedy for various kinds of skin disease. Mr. Jonathan Hutchinson in his "Lectures on Arsenic" (1891) says: "Certainly one of the most remarkable facts as regards the influence of arsenic is that it appears to prevent certain affections which are very similar in nature to those which it causes."

The opinions cited with regard to these drugs are sufficient to warrant a hint of a method which may prove useful. *A priori* we should not have expected the drugs enumerated to have done good in the cases for which they were prescribed—rather otherwise, we should have been disposed to predict harm. But the results obtained were considered satisfactory—in many cases marvellously so.

These physicians speak with great confidence. There is no pessimism about their utterances. They do not bewail the utter uselessness of drugs. These successes standing apart might be looked upon merely as coincidences, as lucky hits by clever physicians, useless as examples for us to follow, but, taken collectively, there is something in them calculated to make us, as the French say, "furiously to think."

True science is made up of facts and of logical deductions therefrom. Here are a few facts. Are they sufficient to warrant the framing of a logical deduction or a generalisation entitled to the weight of a scientific axiom? If so, we might express it thus: "The therapeutic value of a drug corresponds exactly with its pathogenetic, or disease-producing, powers." Or, in other words, "the key to therapeutics is toxicology."

There is a corollary to this dictum, and of equal importance, which deserves more than passing mention. I refer to the small dose. The German physiologist, Max Verworn, in a chapter on "Stimuli and their Actions" ("General Physiology—An Outline of the Science of Life") enunciates the following fundamental biological law, which, he says, has no exception—namely: "Every substance which can paralyse or kill any cell or cell protoplasm can also act in small quantities (on the other side of an indifferent point) as a stimulus to cell activity. The absolute quantities leading to such effects are," he says, "very different with different substances."

Professor Sir W. T. Gairdner expresses exactly the same idea in other words when he says: "Every drug has a therapeutic as well as a physiological action." He does not tell us how to distinguish the dividing line between the two grades of action. He only insists that the therapeutic or stimulating dose must be very much smaller than the physiological or paralysing one. Trousseau, Whitla, Ringer, Murchison, Alfred Meadows, and many others, all suggest the same idea with more or less emphasis.

1. The idea of the energy of infinitesimals, which at one time might have seemed supremely ridiculous, need not henceforward very much shock our imaginations, for can we not now contemplate with equanimity the pathological effects and corresponding therapeutic virtues of the intangible emanations of, say, one-eightieth grain of radium bromide after they have worked a passage through the hard glass of a hermetically-sealed tube—the tube held some distance from the body? These infinitesimal emanations have proved themselves capable of exciting painful and very persistent skin affections.

2. Still further, it has been found that one part of sulphate of copper in 10 to 50 millions of water is sufficient to kill the algæ which frequently become such a nuisance by causing pollution in our public water reservoirs, while a similar extremely dilute solution is very destructive to active typhoid germs. Dare we say the normal cells of the human body would be totally insensible to such contact?

3. So patient and accurate an observer as the late Professor Darwin, in his book on "Insectivorous Plants," referring to the sensibility of the Sun-dew or Venus flytrap, says: "It is an astonishing fact that so inconceivably minute a quantity as one-twenty-millionth of a grain of phosphate of ammonia should induce changes in a gland sufficient to cause a motor impulse to be sent down the whole length of a tentacle, this impulse exciting movements through an angle of about 180°." May not the nervous tissues of the human body be equally sensitive?

4. As a further example may be cited the infinitesimal amount of metallic salts present in colloidal solutions which have lately been so much used by certain French physicians, and the fact that Sir Almroth Wright and Dr. Arthur Latham now recommend for the treatment of tuberculosis doses of one-fifty-thousandth milligram of tuberculin.

The occasion has warranted this passing reference to the high potency of the small dose, as our college education has confirmed in us a natural prejudice in favour of associating energy with mass and weight and quantity. Henceforward if we would keep ourselves abreast of the science of the times this prejudice must be subjected to a considerable amount of judicious modification.

But the main object of this paper is to plead for at least a tentative recognition of the logical deduction: "The therapeutic value of a drug corresponds exactly with its pathogenetic powers."

It is but fair to assert that the distinguished physicians whose good cases we have reviewed might have been guided to such satisfactory results by a conscious recognition of the aphorism just mentioned. In any case, keeping this axiom firmly in mind, having a vivid knowledge of the gross and finer toxic effects of drugs on the human body, with a careful study of the history, pathology, and symptoms of our patients as they come to us, our prescriptions would become simpler.

We should at least have the comfort of working with the support of a valuable rule in accordance with what may shortly be generally acknowledged to be not mere casual coincidence, but natural law. Surely it would be wise for us to focus our attention strongly on this method and work it carefully.

Were the mode of prescribing suggested by this rule more largely recognised and faithfully followed up, empiricism, polypharmacy, stock bottles, heroic dosing, and the prescription of patent medicines at the instigation of enterprising advertising druggists would be quite unnecessary, and would shortly be reckoned as grossly unprofessional as such practice is now universally admitted to be scientifically absurd.

Certainly we should not then have occasion to advertise our hopeless incompetence as physicians, by declaiming that "with two or three exceptions drugs do not cure disease."

